# The aerodynamic behaviour of respiratory aerosols

**DOI:** 10.1186/1753-6561-5-S6-P90

**Published:** 2011-06-29

**Authors:** K Grosskopf

**Affiliations:** 1 College of Engineering, University of Nebraska, Lincoln, USA

## Introduction / objectives

Hospital acquired infections (HAIs) claim on average 90,000 lives each year in the U.S., nearly three times the number of annual highway deaths. Although fewer than 15% of HAIs are directly attributable to airborne transmission, more than a third may be caused by surface microbes aerosolized by the movement of air from building systems, people and equipment. As a result, a study was devised to use a synthetic respiratory aerosol to track the movement airborne contagion with respect to various environmental conditions in a healthcare environment.

## Methods

An actual hospital was used to map the spatial dispersion of synthetic respiratory aerosols with respect to particle size, airflow, door position and healthcare worker movement between a general patient room and corridor.

## Results

Respirable aerosols 0.5µm to <1.0µm were found to exhibit distinctly different aerodynamic behaviours when compared to aerosols 1.0µm -10.0µm. Specifically, aerosols <1.0µm appeared to disperse randomly and uniformly throughout the test space with significantly less regard to mechanical airflow, pressure relationships, door position, and personnel movement when compared to aerosols 1.0µm -10.0µm.

## Conclusion

Since expiratory droplets <1.0µm are believed to be both capable of carrying virus and penetrating into the alveolar region of the lung, these particles may present unique challenges for ventilation systems designed to protect the healthcare population from airborne viral transmission.

## Disclosure of interest

None declared.

